# Optimized Conditions for the Long-Term Maintenance of Precision-Cut Murine Myocardium in Biomimetic Tissue Culture

**DOI:** 10.3390/bioengineering10020171

**Published:** 2023-01-28

**Authors:** Xiaochun Cao-Ehlker, Carola Fischer, Kun Lu, Tobias Bruegmann, Philipp Sasse, Andreas Dendorfer, Roland Tomasi

**Affiliations:** 1 Walter Brendel Centre of Experimental Medicine, University Hospital, Ludwig-Maximilians-Universität Munich, 81377 Munich, Germany; 2 Department of Cardiac Surgery, University Hospital, Ludwig-Maximilians-Universität Munich, 80636 Munich, Germany; 3 Insitute of Cardiovascular Physiology, University of Göttingen, 37073 Göttingen, Germany; 4 German Center for Cardiovascular Research (DZHK), Partner Sites Göttingen and München, 10785 Berlin, Germany; 5 Institute of Physiology I, Life and Brain Center, University of Bonn, 53127 Bonn, Germany; 6 Departement of Anaesthesiology, University Hospital, Ludwig-Maximilians-Universität Munich, 81675 Munich, Germany

**Keywords:** murine heart slices, optical stimulation, electrical stimulation, biomimetic conditions

## Abstract

Organotypic heart slices from mice might provide a promising in vitro model for cardiac research because of the vast availability of genetically modified specimens, combined with the unrestricted feasibility of experimental interventions. However, murine heart slices undergo rapid degeneration in culture. Therefore, we developed optimal conditions to preserve their structure and function in culture. Mouse ventricular heart samples were transversely cut into 300 µm thick slices. Slices were then cultured under various conditions of diastolic preload, systolic compliance and medium agitation. Continuous stimulation was performed either by optical stimulation or by electrical field stimulation. Contractility was continuously measured, and cellular survival, structure and gene expression were analyzed. Significant improvements in viability and function were achieved by elastic fixation with the appropriate diastolic preload and the rapid shaking of a ß-mercaptoethanol-supplemented medium. At 1 Hz pacing, mouse heart slices maintained stable contractility for up to 48 h under optogenetic pacing and for one week under electrical pacing. In cultured slices, the native myofibril structure was well preserved, and the mRNAs of myosin light chain, titin and connexin 43 were constantly expressed. Conclusions: Adult murine heart slices can be preserved for one week and provide a new opportunity to study cardiac functions.

## 1. Introduction

Cardiac research requires structurally and functionally well-preserved experimental models. Several widely used models have their own limitations. For example, isolated cardiomyocytes lack a mechanical load and intercellular communication [1,2]. The intact perfused myocardium provides the native tissue composition; however, the demanding preparation permits only low numbers of experiments [3,4,5,6]. Engineered heart tissue constructed from stem-cell-derived cardiomyocytes exhibits immature properties [7,8,9]. While in vivo animal models are indispensable for studies of basic cardiac biology, their translational potential is being challenged due to a paucity of successful translation from bench to bedside in recent years [10]. However, no in vitro model is currently available to fully recapitulate the whole organism, and investigations on animals are still necessary [11].

Among all models featuring an intact tissue structure, cardiac slices provide the exclusive opportunity for long-term preservation in tissue culture [12,13,14,15,16,17]. When heart tissues are sliced into 300 µm thick layers, not only is the native tissue structure of the myocardium preserved, but this thickness also allows sufficient oxygenation and the supply of nutrients through diffusion [18,19,20,21]. Additionally, from one heart sample, multiple tissue slices can be generated and simultaneously used for a series of experiments, enabling the efficient utilization of experimental materials. Moreover, our group has recently identified biomimetic culture conditions that permit the structural and functional preservation of human heart slices for weeks [21]. Although viable heart slices can be generated with similar techniques from small mammals such as mice, rats and rabbits [14,22,23], conditions permitting successful long-term cultivation have not yet been reported, even though freshly isolated mouse heart slices are vital and exhibit intact cardiac structural and electrophysiological properties [22]. Given the benefits and success of cultured heart slices for cardiovascular research and the feasibility of transgenic mice for studying genes involved in cardiac function/dysfunction, we set out to establish culture conditions to maintain the function and structure of tissue slices from mouse hearts. Since the murine myocardium can be expected to require rapid pacing, and electrical field stimulation is inevitably associated with electrochemical artifacts, we also investigated the optical stimulation of myocardium expressing channelrhodopsin-2 (ChR2). In the past decade, optogenetic techniques have been developed and broadly applied in neuroscience and cardiology [16,24,25,26]. The application of short light pulses can trigger an action potential in cardiomyocytes, thereby inducing contraction [27]. Optical stimulation can minimize possible side effects caused by electrical stimulation but is confined to optogenetically modified cells only. For this reason, we compared optical and electrical stimulation in different biomimetic culture systems.

## 2. Materials and Methods

### 2.1. Mice

All animals were kept in accordance with the Ludwig-Maximilian University of Munich (LMU) guidelines for the care and use of laboratory animals. The animal experiments were carried out in compliance with the animal welfare law and approved by the state agency for environment and agriculture under AZ 55.2-1-54-2532.3-67-13. Transgenic mice, strain CD1 (female, 35–45 g), with the expression of channelrhodopsin-2 [26] were kindly provided by Drs. P. Sasse and T. Bruegmann from the Institute of Physiology I of the University of Bonn. C57/BL6 mice (male, 24–30 g) from Charles River were used for experiments with electrical stimulation.

### 2.2. Surgical Procedure

For experiments with optical stimulation, channelrhodopsin-2 mice were first intraperitoneally injected with 50 μg/kg buprenorphine and 500 U/kg heparin and then killed under deep anesthesia by the rapid excision of the heart through a sternal thoracotomy. Hearts were immediately transferred to a Petri dish with 4 °C slicing buffer (136 mM NaCl, 5.4 mM KCl, 1 mM MgCl_2_, 0.9 mM CaCl_2_, 5 mM HEPES, 0.33 mM NaH_2_PO_4_, 10 mM glucose and 30 mM 2.3-butanedione monoxime). After cardioplegia, both atria were dissected. The intact ventricles were embedded in 4% low-melt agarose (dissolved in a slicing buffer and applied to the tissue at 37 °C) with the apex of the heart facing upward. After the rapid solidification of agarose, the murine hearts were transversely sliced into 300 µm thick slices using a vibratome (VT1200S, Leica Biosystems, Nußloch, Germany), as described earlier [21]. Heart cross-sections (300 μm thick) were glued (Histoacryl, B. Braun Melsungen AG, Melsungen, Germany) at their anterior and posterior walls to small plastic triangles cut from 0.1 mm thick polyester copier clear film (MGW5504, MGW Office Supplies, Tönisvorst, Germany) for organ bath analysis and long-term cultivation in biomimetic culture chambers (BMCCs). For experiments with electrical stimulation, C57/BL6 mice were intraperitoneally injected with 100 µL/10 g of the following drug mixture containing 0.9% NaCl, 10% ketamine hydrochloride, 2% xylazin and 1% acepromazine. The hearts were excised and treated as described for optogenetic mice.

### 2.3. Organ Bath

Freshly prepared or cultured murine heart slices were mounted horizontally in organ baths (Mayflower, Hugh Sachs Electronik (HSE), March, Germany) for the measurement of isometric contraction force, the Frank–Starling mechanism and the force–frequency relationship and perfused with carbon-gassed Krebs–Ringer solution (136 mM NaCl, 5.4 mM KCl, 1 mM MgCl_2_, 0.33 mM NaH_2_PO_4_, 10 mM glucose, 1.8 mM CaCl_2_, 23 mM NaHCO_3_, pH 7.4, 37 °C) at 4 mL/min and field-stimulated at 4 Hz. Pulses of 3 ms duration and an intensity of twofold the excitation threshold were used. The stimulation started immediately after slices were transferred to the organ bath. The maximum contraction force was measured in fresh and cultivated slices under stimulation by the β-agonist isoproterenol, which was applied at a final concentration of 1000 nM to the organ bath. To determine the maximum contraction force and tissue elasticity, the slices were first adjusted to the slack length and then gradually stretched with steps of 0.125 mm every two minutes. Both the active and passive forces were determined within the last ten seconds before an increase in the stretch. With the tearing of the tissue, the experiment was ended.

Drug application experiments with digitoxin were performed in the organ bath after a 30 min equilibration period in the absence of β-stimulation. After the equilibration period, the perfusion was stopped, and digitoxin or the solvent was added. Forces in all experiments were continuously recorded with WinEDR (John Dempster, University of Strathclyde, Glasgow, UK) and analyzed with LabChart 8 Reader (ADInstruments, Dunedin, New Zealand).

### 2.4. Unloaded, Unstirred Culture

Heart slices were cultured on organotypic membranes (PICM0RG50, Merck Millipore, Billerica, MA, USA) [20] in Petri dishes that contained 1 mL of Medium 199 supplemented with penicillin/streptomycin and insulin/transferrin/selenite to guarantee nutrient supply. The slices were immediately placed in a CO_2_ incubator with a temperature of 37 °C and a gas content of 5% CO_2_ and 21% oxygen. Oxygen supply was guaranteed due to the direct contact of the slices’ surfaces with the air. After 24 h, the culture medium was changed, and the culture time was terminated after 48 h.

### 2.5. Submerged, Isometric Culture

Because submerged slices would not adhere to organotypic membranes, they were glued to two plastic triangles perpendicular to the septum and suspended isometrically between two 0.6 mm thick posts before being covered by 3 mL of Medium 199. These conditions enabled the application and generation of preload. To ensure tissue oxygenation, the Petri dishes were rotated horizontally on an orbital shaker (20 rpm) in a standard incubator (37 °C, 0.05 CO_2_, 0.8 humidity). The culture medium was not changed, and the culture was terminated after 48 h.

### 2.6. Optically Stimulated Culture with Preload

To enable effective systolic shortening, the heart slices were elastically suspended between one fixed and one elastic post (0.27 mm tungsten wire) in custom-made culture chambers and were covered with 3 mL of Medium 199 with or without 2-mercaptoethanol. Preload was set to 0.3 mN. The culture chambers were combined on a platform and were placed in a standard incubator (37 °C, 0.05 CO_2_, 0.8 humidity). For conditions of slow medium agitation, the chambers were rotated horizontally on an orbital shaker (20 rpm, PSU-20, Grant Instruments (Cambridge) Ltd., Shepreth Cambridgeshire, UK), while rapid shaking was produced by 12° tilting at 60 min^−1^ (ST5 CAT, Zipperer-GmbH, Wertheim, Germany). To perform optical stimulation, light-emitting diodes (LEDs, CREE XP-EM2, 475 nm, LED-TECH.DE optoelectronics GmbH, Moers, Germany) were centrally placed at a 5 mm distance underneath each culture chamber of the biomimetic culture system. Pulses of 3 ms duration and currents between 0.1 and 1.2 A were used. The stimulation threshold was defined as the current required to induce 50% of the maximum contraction force in each slice. The thresholds for detectable minimum and maximum contractions were determined by pausing the stimulation for 4 min, followed by gradual increases in the LED current every 2 min from 8.8 mA to 1 A. Throughout the culture period, the pulse current was set to 1 A, corresponding to a luminous flux of 80 lumens. The LED specifications indicated a linear relationship between the driving current and emission intensity. The stimulation frequencies were set at 0.1 Hz, 0.5 Hz and 2 Hz. Again, the culture was terminated after 48 h.

### 2.7. Electrically Stimulated Culture with Preload

The system for electrical biomimetic cultivation was based on the elastic mounting of tissue slices and on the principle of continuous force measurement by assessing the displacement of a small magnet located at the tip of the elastic wire, as previously described [21]. Specifically, murine heart slices were elastically (spring constant 14 N/m) suspended between two posts within custom-made biomimetic culture chambers (BMCCs). BMCCs were combined on biomimetic platforms with integrated electronic control and were placed in a standard incubator (37 °C, 0.05 CO_2_, 0.8 humidity). The medium was continuously agitated by tilting the platform around the BMCC transversal axis (60 min^−1^, 12° tilting angle, ST5 CAT, M. Zipperer GmbH, Ballrechten-Dottlingen, Germany). Slices were cultured in Medium 199 supplemented with penicillin/streptomycin, insulin/transferrin/selenite and 2-mercaptoethanol (50 µM). Part of the medium was exchanged (1.6 mL of 2.4 mL total volume in each BMCC) at 36–48 h intervals. A preload of 0.3 mN was applied at the beginning of the cultivation and adjusted every 12 h within the first 24 h if necessary. Pacing was performed at 1 Hz with bipolar 50 mA pulses consisting of 1 ms charging and discharging pulses separated by a 1 ms interval. The unphysiological low beating rate was chosen in order to keep the demands for O_2_ and nutrients at a minimum.

### 2.8. Force Measurement and Analysis

The contraction force of elastically mounted slices was continuously recorded as described previously [21]. During biomimetic culture, preload and contractility were derived from the deflection of the elastic spring wire and were recorded continuously. The tonic contracture that eventually occurred at the start of incubation was quantified as the spontaneous increase in diastolic tension over 20 min. The recorded contraction curves were imported and analyzed with “LabChart Reader” (ADInstruments, Dunedin, New Zealand) to determine diastolic tissue tension and the systolic force generated by the murine slices during a cultivation period of 48 h for optical stimulation and 7 days for electrical stimulation.

### 2.9. Viability Determination

A total of 0.5 mg/mL methylthiazolyldiphenyl-tetrazolium bromide (MTT) was added to both fresh and cultured heart slices and incubated at 37 °C for 20 min. The water-soluble tetrazolium salt of MTT is reduced to water-insoluble formazan in the presence of NADP. Thus, the violet color of formazan represents live cells [28]. The MTT-positive area was examined by brightfield microscopy and was analyzed with Image J. The ratio of the viable area to the total area of the slice was calculated.

### 2.10. Confocal Microscopy

Immunofluorescence of murine heart slices was performed as described before [21]. In brief, fresh and cultured slices were fixed with 4% formaldehyde at 4 °C for 24 h, equilibrated with a sucrose gradient and permeabilized in 1% Triton-X at 4 °C overnight. The slices were then blocked with 3% BSA at 4 °C overnight and incubated with primary antibodies (rabbit anti-connexin 43, C6219, Sigma; mouse anti-actinin, A7811, Sigma-Aldrich, Saint Louis, MO, USA) at 4 °C overnight, followed by secondary antibodies (anti-Rabbit Alexa 488, anti-mouse Alexa 546) and TO-PRO3 (excitation 633 nm, emission 660 nm) for nuclear staining. The samples were analyzed using a confocal laser scanning microscope (Leica SP8X WLL, Leica Microsystems, Wetzlar, Germany).

### 2.11. Real-Time qPCR

RNA extraction was performed using the RNAeasy Mini Kit (Qiagen, Hilden, Germany). RNA concentrations were measured using a NanoDrop 2000 spectrophotometer (Thermo Fisher Scientific, MA, USA). Equal amounts of RNA were used to generate cDNA samples by reverse transcription using a cDNA Synthesis Kit (Roche, Basel, Switzerland) following the manufacturer’s instructions. All qRT-PCR analyses were performed in duplicate on a LightCycler 1.5 (Roche, Basel, Switzerland) Detection System using Power SYBR Green PCR Master Mix (Invitrogen, MA, USA). Specifically, designed primers (listed in Table 1) were synthesized by Eurofins Genomics (Ebersberg, Germany). Cycling conditions were as follows: initial denaturation: 95 °C for 5 min, 45 cycles at 95 °C for 10 s, 60 °C for 30 s, and 72 °C for 15 s. β-Actin was used as a reference gene in all experiments. In addition, mRNA expression was referenced to total RNA. The relative mRNA expression of target and reference genes was calculated using the Roche LightCycler Quantification Software 3.5.

### 2.12. Statistical Analysis

All data were analyzed using GraphPad Prism 7.0 (GraphPad Software Inc., San Diego, CA, USA). Differences between two groups were evaluated using t-tests. For more than two groups, one-way ANOVA with post hoc test was used. The statistical significance level was set at *p* < 0.05 for both tests. In the figures, experimental data are presented as means ± standard error of the mean (SEM).

## 3. Results

### 3.1. Diastolic Tension Is Critical for Tissue Integrity

**Figure 1:**
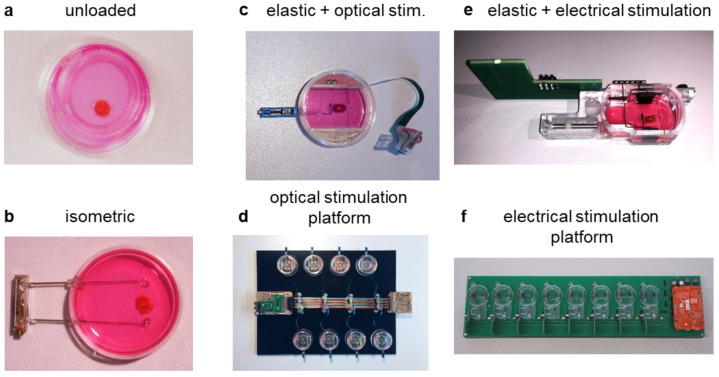
Implementation of various conditions of biomimetic culture: (**a**) Unloaded tissue slice attached to organotypic membrane. (**b**) Isometric fixation of cultured slice between two fixed posts. (**c**–**f**) Elastic mounting of cultured slice to spring wires. (**c**,**e**) Incubation chambers suitable for either optical or electrical stimulation. (**d**,**f**) Microcontroller platforms for optical or electrical stimulation combined with continuous force measurement.

**Figure 2:**
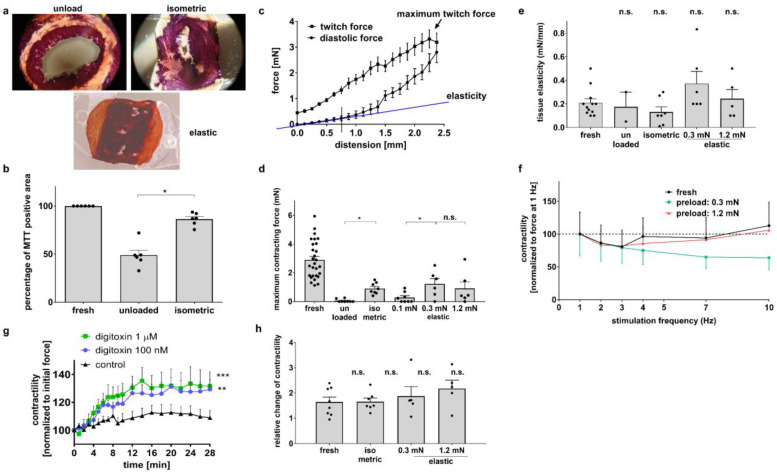
Effect of various culture conditions on preservation and performance of murine ventricular slices (fresh = no cultivation; all others: cultured for 48 h): (**a**) MTT staining of slices after unloaded, isometric or elastic culture. Vital cells are stained in purple color. (**b**) Statistical analysis of heart slice viability after 2 days of culture under various conditions (n = 5, mean ± SEM, * *p* < 0.05). (**c**) Frank–Starling relationship in fresh murine heart slices determined in an organ bath (n = 4, mean ± SEM). (**d**) Maximum twitch force of slices after 2 days of culture under various conditions, measured in an organ bath (n = 6–28, mean ± SEM, * *p* < 0.05, n.s. not significant). (**e**) Tissue elasticity of slices cultured for 2 days under various conditions (n = 2–12, mean ± SEM, n.s. not significant). (**f**) Force–frequency relationship (n = 2–4, normalized to 1 Hz) after 48 h after slicing. (**g**) Demonstration of the positive inotropic effect of digitoxin using the murine slice model (n = 6, mean ± SEM, * *p* <0.05, *** *p* <0.001, ** *p* <0.01). (**h**) Relative change in slice contractility after stimulation with 1 µM isoproterenol (n = 5–8, mean ± SEM, n.s. not significant).

In order to carry out long-term cultivation, we first established optimal culture conditions to maintain the structure and function of murine heart slices in the absence of stimulation and rhythmic contractions. Initially, slices were cultured for 48 h on organotypic membranes, referred to as the unloaded condition (Figure 1a). Under these conditions, both low cell viability (Figure 2a,b) and significant functional loss (Figure 2d) were observed. To improve tissue integrity, slices were suspended between two fixed posts, referred to as the isometric condition (Figure 1b). Cell viability after culture significantly increased (Figure 2a,b), and in organ bath measurements, the maximum contraction force was maintained at a significantly higher level than that of unloaded slices (Figure 2d). Next, to approach auxotonic contractions and to analyze the optimal diastolic tissue tension, slices were suspended between a movable and an elastic post with a spring constant of 4 N/m, allowing the application of various preload levels to the slices. This condition is referred to as the elastic condition (Figure 1c–f). Cell viability was maintained under these culture conditions (Figure 2a). Figure 2c shows the Frank–Starling relationship in fresh mouse heart slices in the organ bath. Based on the Frank–Starling relationship, the maximum contraction force and tissue elasticity could be calculated. Although the maximum contraction force also decreased under elastic conditions, preloads of 0.3 mN and 1.2 mN maintained the maximum contraction force at 50% and 30% in fresh slices, respectively (Figure 2d). These results suggest that a preload of at least 0.3 mN and the elastic suspension of the myocardium is crucial for the tissue preservation of murine heart slices in culture. Tissue elasticity under all conditions was not significantly different compared with fresh slices, indicating no contracture or degradation during culture (Figure 2e). Next, in the organ bath, we analyzed the force–frequency relationship in fresh slices and under elastic conditions with 0.3 mN and 1.2 mN preloads. In all slices, contraction force decreased when electrical pacing was accelerated from 1 Hz to 3 Hz (Figure 2f). At higher heart rates, the contraction force of fresh and 1.2 mN preloaded slices increased compared to the force of slices cultured at 0.3 mN preload, which further decreased (Figure 2f). To show that murine heart slices are suitable for functional experiments, we treated the slices in the organ bath with two different concentrations of digitoxin (100 nM and 1µM) and compared the effects with a control group. The contractility of treated mouse heart slices increased significantly (Figure 2g), with only a small additional effect of the higher digitoxin concentration compared to the lower concentration. In order to demonstrate that cultivated and fresh slices respond in a similar way to an exemplary drug treatment, we stimulated the contractility of fresh and cultivated slices with isoproterenol and found no differences in the relative change in twitch force (Figure 2h). The results suggest that elastic conditions and a preload of at least 0.3 mN are needed to preserve the structure and function of murine heart slices within the first 48 h to a certain extent. In addition, it appears that drug experiments are feasible with cultured murine heart slices.

### 3.2. Optical Stimulation Impairs the Preservation of Myocardium after 48 h Culture

**Figure 3:**
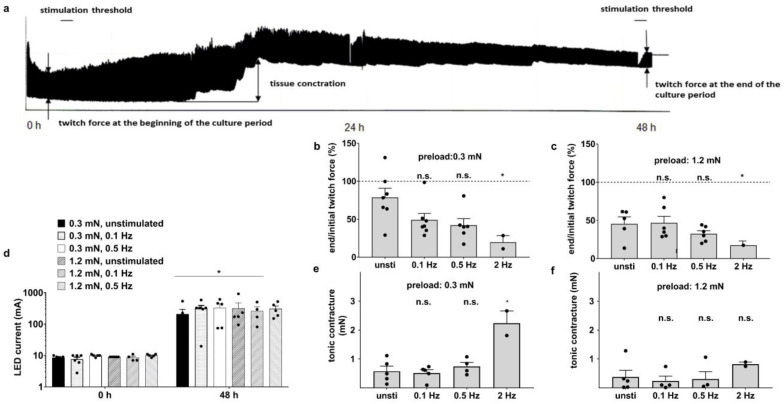
Effect of optical stimulation on elastically mounted myocardium during 48 h cultivation. (**a**) Representative recording of twitch force of a mouse heart slice during the culture period in a biomimetic culture chamber. (**b**,**c**) Twitch force alteration after culture of optically stimulated and unstimulated heart slices with various stimulation frequencies and different preloads (n = 2–5, mean ± SEM, * *p* < 0.05, n.s. not significant). (**d**) Stimulation thresholds at the beginning and at the end of the culture period (n = 4–5, mean ± SEM, * *p* < 0.05). (**e**,**f**) Tonic contracture of optically stimulated and unstimulated heart slices with various stimulation frequencies and different preloads (n = 3–5, mean ± SEM, * *p* < 0.05, n.s. not significant).

The continuous stimulation of murine heart slices is desired for systolic force measurement, mechanical and metabolic loads, excitation and electrical activity. A representative recording of twitch force for 48 h in the biomimetic culture system under optical stimulation is shown in Figure 3a. A significant decrease in twitch force, occasional tissue contracture and an increase in stimulation threshold occurred during culture. To analyze these observations in more detail, further experiments were performed in biomimetic culture chambers (BMCCs) using slices with different diastolic preloads (0.3 mN and 1.2 mN) and various optical stimulation frequencies (0.1 Hz, 0.5 Hz and 2 Hz) throughout the 48 h cultivation period. As a control group, slices with 0.3 mN and 1.2 mN diastolic preloads were cultured without continuous optical stimulation. To measure twitch force in these slices, they were stimulated for 1 h at the beginning and at the end of the cultivation period. Compared to the initial twitch force, the contractility decreased within 48 h in all culture conditions (Figure 3b,c). In addition, the twitch force of slices with a higher stimulation frequency (2 Hz) was significantly reduced compared to unstimulated slices (Figure 3b,c). Stimulation thresholds were measured at the beginning and the end of the culture period. Light intensity had a linear relationship with the LED current. At the beginning of the cultivation period, the stimulation threshold was 8.8 mA, and at the end, it increased to an LED current of 300 mA (Figure 3d). In addition, incidental increases in diastolic forces were observed in all slices (Figure 3e,f), reflecting partial contracture. Slices with a preload of 0.3 mN developed significantly increased diastolic contracture with a stimulation frequency of 2 Hz (Figure 3e) compared to slices with a preload of 1.2 mN (Figure 3f). Tissue contraction and stimulation threshold increments indicate irreversible damage to slices under optical stimulation within 48 h.

### 3.3. Cellular Structure Is Preserved in Biomimetic Culture under Optical Stimulation

**Figure 4:**
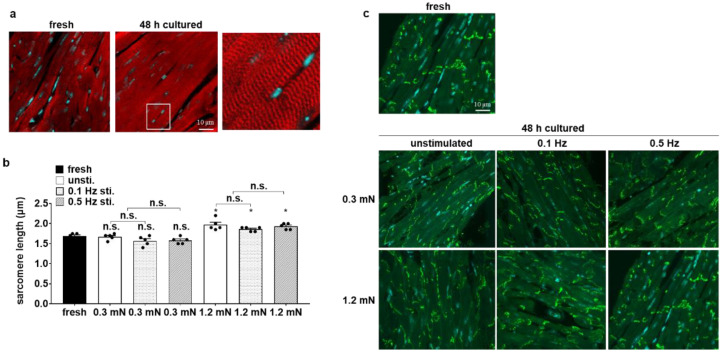
Maintenance of tissue structure in biomimetic culture. (**a**) Sarcomere alignment in both fresh and cultured slices with a preload of 0.3 mN. Myofibrils are stained in red, and nuclei are stained in blue. (**b**) Cardiomyocyte sarcomere length under various culture conditions (n = 5, mean ± SEM, * *p* < 0.05, n.s. not significant). (**c**) Distribution of connexin 43 within slices under different culture conditions after 48 h. All slices are stained with anti-connexin 43 antibodies (green) and Topro 3 (blue) for nuclei.

Confocal microscopy was utilized to analyze cellular structures in cultured slices, including the myofibril arrangement, length of sarcomeres and connexin 43 distribution. Sarcomere alignment was not different between fresh and cultured slices (Figure 4a). We found no differences between the sarcomere length in fresh slices (1.69 μm ± 0.06) and that in slices with 0.3 mN preload (1.61 μm ± 0.09), whereas the sarcomere length was higher in slices with 1.2 mN preload (1.92 μm ± 0.10; Figure 4b). Due to the increased stimulation threshold of the heart slices (Figure 3d), we next analyzed the distribution of connexin 43 in cultured slices. Connexin 43 is the primary component of gap junctions in adult mice and plays an essential role in the synchronized contraction of the heart [29,30]. In all slices, connexin 43 was concentrated at the intercalated discs, a typical pattern in cardiomyocytes (Figure 4c), indicating that there was no redistribution of connexin 43 during cultivation.

### 3.4. Medium Agitation and Antioxidant Supplement Improve Contractility under Optical Stimulation

Since the poor resistance of heart slices to continuous stimulation may be related to higher metabolic demands and oxygen consumption, we set out to improve oxygen availability and to test an antioxidative medium supplement.

**Figure 5:**
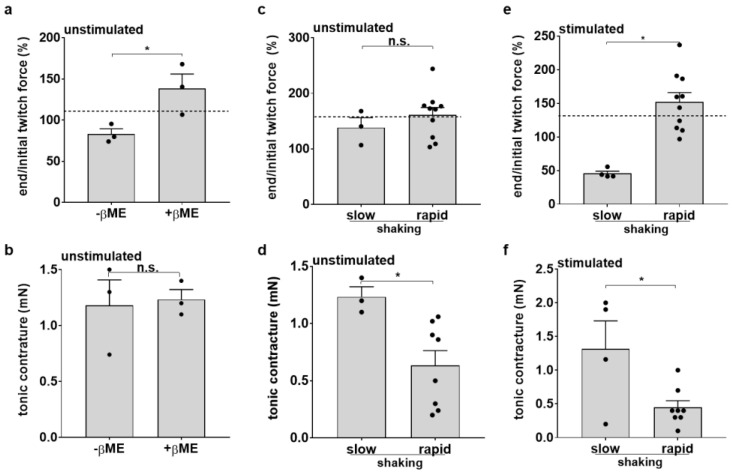
Interventions to improve biomimetic culture. (**a**,**b**) Effects of β-mercaptoethanol supplementation of the culture medium on twitch force (**a**) and tonic tissue contracture (**b**) in unstimulated, elastically mounted slices (n = 3, mean ± SEM, * *p* < 0.05, n.s. not significant). (**c**–**f**) Effects of high-intensity medium agitation (rapid shaking) on the maintenance of twitch force (**c**,**e**) and occurrence of tonic tissue contracture (**d**,**f**) during biomimetic culture of unstimulated (**c**,**d**) and optically stimulated (**e**,**f**) slices (n = 3–9, mean ± SEM, * *p* < 0.05, n.s. not significant).

To prevent cellular oxidative stress, β-mercaptoethanol (β-ME, 50 µM) was added to unstimulated slices at the start of the culture period. Twitch force was significantly higher after 48 h in culture in the presence of β-ME (138% ± 17.71 vs. 83% ± 6.4) (Figure 5a). β-ME could not prevent diastolic contracture (Figure 5b). Our results indicate a protective effect of β-ME for the slices, and thus, in all following experiments, this antioxidative drug was added to the culture medium. To improve the oxygen supply to the slices, we compared the effects of slow versus rapid shaking. Although the rapid tilting of unstimulated slices around their transversal axes did not significantly improve twitch force (Figure 5c), the tonic contracture of the tissues was significantly decreased (Figure 5d). We next investigated these interventions in optically stimulated slices. Indeed, the twitch force was higher under fast tilting conditions and with the addition of β-ME in the culture medium (Figure 5e). Consistently, tonic contracture was significantly decreased (Figure 5f). In summary, the results indicate the improved functional preservation of optically stimulated mouse heart slices with faster tilting of the platform and the addition of β-ME in the culture medium.

### 3.5. RNA Expression Indicates Downregulation of Sarcomeric Proteins and Presence of Hypoxia

To analyze the molecular mechanism of functional decay under optical stimulation, we performed real-time quantitative RT-PCR to detect the mRNA expression of genes involved in cardiovascular function and structure. The genes analyzed in our study were cardiac myosin light chain 2 (MLC-2V), titin (N2A and N2B), connexin 43 (GJA1) and glucose transporter 1 (Glut1). Table 2 presents the ratios of mRNA expression in selected groups subjected to different culture conditions. A comparison of cultured slices with fresh tissue revealed the consistent suppression of MLC-2V and N2B expression after the cultivation period, whereas the Glut1 transcript was greatly enhanced. In general, the expression levels of these genes yielded higher numbers when they were referenced to the total amounts of extracted mRNA (Table 2B), thus indicating the upregulation of β-actin expression during the culture period. As a consequence, no consistent influence of tissue culture on N2A and GJA1 mRNA expression can be stated. The regulation of β-actin expression was not evident in the various groups of cultured tissues. As such, a significant reduction in Glut1 expression could be accomplished with intense, as compared to slow, medium agitation. As expected, the results confirm structural damage due to hypoxia in slices in the first experiments, with a reduction in hypoxia with the faster tilting of the platform.

### 3.6. Electrical Stimulation Preserves Full Contractility for Days

**Figure 6:**
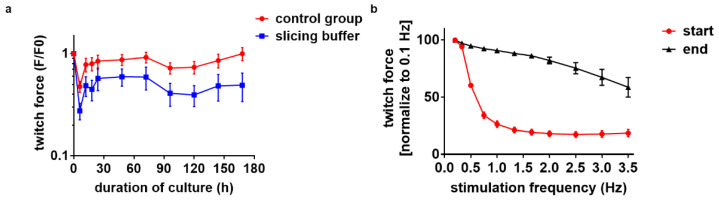
Long-term maintenance of murine heart slices under electrical stimulation. (**a**) Twitch force registration for 168 h of electrically stimulated heart slices with and without treatment with slicing buffer for 1 h at the beginning of culture in advanced biomimetic culture chambers (23 slices of 5 independent preparations for the control group; 9 slices of 2 independent preparations for the slicing buffer group). (**b**) Force–frequency relationship in slices at the beginning and at the end of culture (n = 3).

Since the function and structure of mouse heart slices were only partially preserved during 48 h in our first BMCCs, and the optical stimulation of genetically modified myocardium provided no benefits, we next investigated the effect of the electrical stimulation of wild-type mouse heart slices in BMCCs (Figure 1f). Up to eight vital 300 µm thick slices could be prepared from each mouse heart (n = 5). The slices were cultivated under elastic conditions with a preload of 0.3 mN and were electrically stimulated immediately after their transfer to the BMCCs. Pacing was performed at 1 Hz with bipolar 50 mA pulses comprising 1 ms charging and discharging pulses separated by a 1 ms interval. Again, slices were cultured in Medium 199 supplemented with penicillin/streptomycin, insulin/transferrin/selenite and β-ME (50 µM). Preload was readjusted after 1 h of cultivation and was not further manipulated throughout the subsequent culture period. With these conditions, the contraction force of the slices was preserved for up to 7 days (Figure 6a). The force–frequency relationship was negative within the first day but showed a positive trend after 1 week of culture (Figure 6b). With the goal to reduce the initial contracture and to allow gradual adaptation, we applied a mixture of 1 mL of slicing buffer and 1.4 mL of culture medium during the first hour of cultivation. We found no additional benefit of this intervention but rather the weaker twitch force of slices after this presumably protective treatment (Figure 6a).

## 4. Discussion

Cardiovascular diseases necessitate research to discover and test novel preventive and curative therapies. A native and complex tissue structure, such as those present in animal models, is a frequent prerequisite for the assessment of disease mechanisms. In trying to reduce the number of animals for cardiovascular research, we established a new in vitro model, with which we were able to generate up to eight slices from one mouse heart that can be cultivated in custom-made BMCCs for up to 7 days. Our biomimetic platform allows the elastic stretching of the slices and continuous electrical stimulation. The mechanical stretching of tissue slices is necessary for maintaining tissue integrity because viability and contractility were largely impaired in unloaded slices (Figure 2a,b,d,e). In accordance with our results, the application of preload has been reported by others to improve heart tissue organization [15]. Since the myocardium is exposed to wall tension caused by passive ventricular filling in diastole and active contraction in systole, cardiomyocytes degenerate in the absence of load [31]. With our arrangement of biomimetic tissue culture, a preload of 0.3 mN appeared appropriate to approach physiological conditions, since an identical length of 1.6 µm was determined in fresh slices (Figure 4b). Obviously, this value does not match the sarcomere length of 2.0 µm that has been observed in the native and intact mouse myocardium [32]. However, we measured the sarcomere length in unloaded, PFA-treated slices, which are typically subject to shrinkage during fixation. Consistent with this consideration, a preload of 1.2 mN increased the sarcomere length to 1.9 μm, but did not further enhance contraction force (Figure 2d). The assumption that a preload of 0.3 mN is close to optimal conditions for force development is further corroborated by the estimation of the corresponding wall stress. Assuming a wall thickness of 1.5 mm and a radius of 2 mm in the mouse heart [33], the cross-sectional area of a myocardial slice can be calculated as 2 × 1.5 × 0.3 = 0.9 mm^2^. Consequently, a preload of 0.3 mN will produce a diastolic wall stress of 0.3/0.9 = 0.39 mN/mm^2^. This corresponds well to the physiological diastolic wall stress of the murine myocardium that is generated by a transmural pressure of 0.65 mN/mm^2^ [34]. The application of LaPlace’s law for hollow bodies [35] results in a calculated wall stress of 2 × 0.65/1.5/2 = 0.33 mN/mm^2^. In addition, the relationship between systolic force and the end-diastolic volume in cardiac muscle is based on length-dependent changes in myofilament calcium sensitivity. Since there was no significant difference in twitch force between slices with preloads of 0.3 mN and 1.2 mN (Figure 2d), it seems that mouse heart slices may have an altered calcium sensitivity [36]. Therefore, and because there were no differences in the gene expression of titin isoforms in particular between the two preload values, we decided to proceed with a preload of 0.3 mN in further experiments. For the mechanical characterization of the slices, all experiments in the organ bath were performed in the presence of 1000 nM isoproterenol to achieve the maximum contraction force during the experiments. The maximum contraction force developed in the organ bath was significantly reduced in all cultured slices compared to fresh ones (Figure 2d). Together with the decreased gene expression of myosin light chain 2 (MLC2), these results implied that tissue remodeling occurred during cultivation, which was also observed in human heart slices [20]. In contrast, we found no changes in the tissue elasticity of the slices (Figure 2e), even though the mRNA expression of titin (N2A, N2B) was reduced in cultured slices (Table 2). The tissue elasticity of myofibrils depends above all on the amount of titin and extracellular matrix, particularly collagen [37]. The amount of collagen was not measured in this study. To further approach physiological biomimetic conditions, the continuous stimulation of cultured slices was implemented. Optical stimulation was primarily addressed because it is not hampered by the electrochemical reactions of electrical stimulation, which include the generation of oxygen radicals and H^+^ due to the oxidation of Cl^−^ and H_2_O [38,39]. Optical stimulation led to defined contractions of the tissue slices, but twitch force decreased over time, and the stimulation threshold increased significantly within the first 48 h of culture (Figure 3b–d). In contrast, unstimulated slices maintained their twitch force at a higher level (Figure 3b,c) compared to the stimulated ones. As a possible reason for desensitization to optical stimulation, we tested the coupling of cardiomyocytes via gap junctions. The connexin 43 distribution and mRNA expression did not differ between stimulated and unstimulated slices (Figure 4c). Since the induction of Glut1 expression provided clear evidence for hypoxia, the inhibition of gap junction conductivity by dephosphorylation [40] would be a likely mechanism of uncoupling. It should also be considered that optical stimulation will be confined to the surface layer of the highly light-absorbing tissue, so the direct phototoxicity of the excitation light would also arouse the phenomenon of desensitization. Both hypoxia and phototoxicity are possible mechanisms for the faster deterioration of contractility performance at higher beating rates (Figure 3b,c). In order to minimize the oxygen consumption of the slices, we did not apply the physiological pacing rate of up to 10 Hz for the murine heart [41], but only subphysiological frequencies between 0.1 Hz and 2 Hz. Even though a slice thickness of 300 μm has been shown to allow sufficient oxygen diffusion to the central portion of the slices [19], we could not rule out the possibility that optical stimulation causes higher oxygen demand, and thus, oxygen depletion in the center of the tissue slices might affect their functional integrity. Insufficient oxygen supply impairs various metabolic processes in tissue slices, leading to functional loss during the course of cultivation [20]. Consistent with the loss of twitch force, the expression level of myosin light chain 2 largely decreased (Table 2). We hypothesized that the oxygen distribution in the medium was a limiting factor of oxygen availability, and therefore, we intensified medium agitation to meet the higher oxygen demand. With the rapid tilting of the BMCCs, the stimulated slices displayed significantly better functional integrity (Figure 5e), whereas the contractility of unstimulated slices remained unaltered (Figure 5c). Consistently, glucose transporter 1 expression levels were increased to a lesser extent in rapidly tilted slices compared to slowly tilted ones, confirming myocardial hypoxia in the latter condition (Table 2) [42]. Along with hypoxia, oxidative stress is a major threat to living cells by generating cell-damaging reactive oxygen compounds. Glutathione plays a critical role in protecting against various damaging radicals [43], particularly in stimulated heart tissue slices. The addition of β-mercaptoethanol to the culture medium has been shown to enhance glutathione synthesis in bovine embryos [44]. Indeed, twitch force tended to be better maintained when β-ME was present in the culture medium (Figure 5a). It seems that tissue damage caused by reactive oxygen species may be an additional cause of the functional loss of the tissue slices. No effect of β-ME on the diastolic contracture of the slices was seen within the first 48 h of culture (Figure 5b). Diastolic hypercontracture causes irreversible cell shortening [45,46]. It has been reported that calcium overload in cardiomyocytes leads to hypercontracture [47,48]. Several conditions can cause calcium overload, such as hypoxia, ischemia, hypertrophy, oxidative stress and heart failure [49,50]. In myocardial mouse slices, hypoxia was associated not only with the loss of contractility but also with occasional diastolic contracture.

Finally, we wanted to figure out whether the detrimental effect of optical stimulation is generally related to the enhanced activity and oxygen demand of the myocardium, or if it reflects a peculiar consequence of light exposure. We therefore tested electrical stimulation with the consideration of all conditions optimized for continuous activity. An advantage of electrical stimulation is the fact that it can be universally applied to all excitable tissues and does not depend on transgenic modifications. Optimized conditions, such as rapid tilting and the addition of β-ME to the culture medium, were adopted for the modified platform, and the method of anesthesia of the mice was changed. Instead of anesthesia with isoflurane, the mice were intraperitoneally anesthetized, eventually taking advantage of the fact that the combination of ketamine and xylazine showed the highest cardiac depression in echography in healthy mice [51]. With electrical stimulation, we achieved stable culture conditions for up to 7 days with the preservation of a twitch force comparable to fresh slices (Figure 6a). The force–frequency relationship was negative over the low-frequency range (Figure 6b), as described before [52]. The FFR over the frequency range close to the physiological heart rate is positive and qualitatively similar to that in larger mammalian species, although the positive FFR is less prominent [52]. Next, to allow the gradual adaptation of the slices, we added a slicing buffer within the first hour of cultivation to the medium. We found no additional benefit of this intervention but rather a weaker twitch force compared to slices without protection (Figure 6a).

## 5. Conclusions

In summary, we have been able to preserve the viability and contractility of mouse heart slices in BMCCs for up to 7 days and propose such tissue cultures as an alternative in vitro model for cardiac research with the advantage to reduce animal testing. As the first validation tests, the drug treatment of fresh slices with digitoxin and of fresh and cultivated slices with isoproterenol showed a stable contractility response and no relevant differences between fresh and cultivated slices. It may be conceded that a detailed evaluation of experimental protocols is still required to fully develop this potential.

## 6. Patents

A patent describing the relevant principles of magnetic force measurements has been granted to AD under the registration Nr. EP3383995B1.

## Figures and Tables

**Table 1:** RT-PCR primers (MLC-2V: myosin light chain 2; N2A and N2B: titin; GJA1: connexin 43; GLUT1: glucose transporter 1).

Gene	Forward Primer	Reverse Primer
MLC-2V	ATCGACAAGAATGACCTAAGGGA	ATTTTTCACGTTCACTCGTCCT
GJA1	ACAAGGTCCAAGCCTACTCCA	CCGGGTTGTTGAGTGTTACAG
N2A	GGCATCTCCAGGACGTTACTC	TTCACTCTGCCTTGAGGTTTAAG
N2B	GCACAGAAGGAAGATCCTGA	ACCTGCTTTTCCTCAAGTGCT
Glut1	CAGTTCGGCTATAACACTGGTG	GCCCCCGACAGAGAAGATG

**Table 2:** Changes in mRNA expression of cardiac genes after 48 h cultivation under various conditions. Tissue slices were cultivated with 0.3 or 1.2 mN preloads and in the absence or presence of optical stimulation at 0.5 Hz and β-mercaptoethanol (β-ME, 50 µM). Expression of individual genes was measured with real-time PCR and was normalized to that of (A) β-actin or total mRNA (B). Values correspond to ratios of mRNA abundance in selected pairs of culture conditions (n = 7–12, *t*-test * *p* < 0.05, stim. = stimulated, unstim. = unstimulated).

A: Gene Expression Referenced to β-actin mRNA					
Culture condition	MLC-2V	N2A	N2B	GJA1	Glut1
0.3 mN unstim. slices/fresh	0.068 *	0.207 *	0.063 *	0.404 *	3.149 *
0.3 mN stim. slices/fresh	0.055 *	0.19 *	0.055 *	0.402 *	3.574 *
1.2 mN/0.3 mN (stim. slices)	1.090	1.111	1.276	NA	0.785
rapid shaking/ slow shaking (stim.+unstim. slices)	1.022	0.858	0.663	1.176	0.426 *
+ß-ME/-ß-ME (stim.+unstim. slices)	1.043	1.106	1.011	1.001	1.057
**B: Gene expression referenced to total RNA**					
**Culture condition**	**MLC-2V**	**N2A**	**N2B**	**GJA1**	**Glut1**
0.3 mN unstim. slices/fresh	0.37 *	1.12	0.341 *	2.184	16.994 *
0.3 mN stim. slices/fresh	0.324 *	1.108	0.324 *	2.341	20.78 *
1.2 mN/0.3 mN (stim. slices)	0.928	0.946	1.087	N/A	0.668
rapid shaking/ slow shaking (stim.+unstim. slices)	1.059	0.889	0.687	1.218	0.442 *
+ß-ME/-ß-ME (stim.+unstim. slices)	1.054	0.995	1.088	1.098	1.040

## Data Availability

All data supporting the findings of this study are available from the corresponding authors on reasonable request.
